# Dolutegravir resistance in three pregnant and breastfeeding women in South Africa

**DOI:** 10.4102/sajhivmed.v24i1.1531

**Published:** 2023-11-28

**Authors:** Ninke Fourie, Kate Rees, Denis Mali, Bridget Mugisa, Cara O’Connor, Natasha Davies

**Affiliations:** 1 Anova Health Institute, Johannesburg, South Africa; 2 Department of Community Health, School of Public Health, Faculty of Health Sciences, University of the Witwatersrand, Johannesburg South Africa; 3 United States Agency for International Development, Pretoria, South Africa

## Introduction

Dolutegravir (DTG) has superior efficacy, safety, tolerability, and a better resistance profile compared with non-nucleoside reverse transcriptase inhibitors (NNRTI), making it the preferred antiretroviral therapy (ART) choice for adults and adolescents, including pregnant and breastfeeding women (PBFW).^1,2,3^ Dolutegravir became widely available in South Africa in 2019, when tenofovir/lamivudine/DTG (TLD) was adopted as the preferred first-line regimen.

Several studies have shown that even with pre-existing nucleoside reverse transcriptase inhibitor (NRTI) resistance, switching to DTG, while maintaining the same NRTI backbone, achieves viral suppression rates of over 90%.^4,5,6^ The ADVANCE trial demonstrated that DTG-containing regimens successfully re-suppress 95% of people experiencing viral load (VL) rebound above 1000 copies/mm^3^, without switching drugs.^7^ These trials informed South Africa’s 2023 national HIV management guidelines, which recommend a very high threshold for resistance testing. Any patient with a VL of > 50 copies/mm^3^ requires enhanced adherence support with VL monitoring. When a patient has met the criteria for virological failure, defined as two consecutive VLs > 1000 copies/mm^3^, resistance testing is recommended, under specific conditions.^8^ These conditions include: previous confirmed ART regimen failure (known as TLD2, or second-line TLD); at least two years on TLD; and adherence of > 80% by objective measurement. If a client is on TLD with no record of ART failure (known as TLD1), resistance testing is only indicated in special circumstances.^8^

However, within programmatic settings, reports of DTG resistance are emerging, including a 5.8% rate of DTG-resistant mutations among adolescents and young adults in a Tanzanian national survey.^9^ A recent article also described South Africa’s first published case of DTG resistance, in a treatment-experienced, integrase strand inhibitor (InSTI)-naïve adolescent.^10^

The use of antiretroviral drugs that rapidly and safely achieve and sustain maternal viral suppression during pregnancy and breastfeeding is essential for vertical transmission prevention (VTP).^11^ Between 2010 and 2022, implementation of VTP has led to a dramatic 58% decline in new HIV infections among children under five years of age.^12^ In South Africa, the most recent vertical transmission rate at 18 months of age is 4.3%.^11^ The roll-out of DTG has offered hope for further reducing vertical transmission, since the time to viral suppression is approximately halved by DTG when compared to the previously preferred first-line antiretroviral, efavirenz (EFV).^13^ Emerging DTG resistance will pose challenges to effective VTP. With increased frequency of resistance, there is increased risk for adverse maternal outcomes and vertical transmission of resistant HIV. Managing infants who acquire a resistant virus can be especially difficult given limited access to more tolerable and effective ART options for paediatric populations.

To our knowledge, this is the first clinical case series describing confirmed DTG resistance in PBFW in a programmatic setting.

## Cases

### Data collection

Each patient included in this case series was managed directly by two of the authors, Fourie and Davies, within advanced clinical care or higher risk pregnancy clinics. The patients provided written consent to be included in the case series. A full file review, extensive search of the National Health Laboratory Services (NHLS) test results database, review of pharmacy ART pick-ups, and in-depth interviews with each woman were conducted.

### Case 1

**FIGURE 1:**
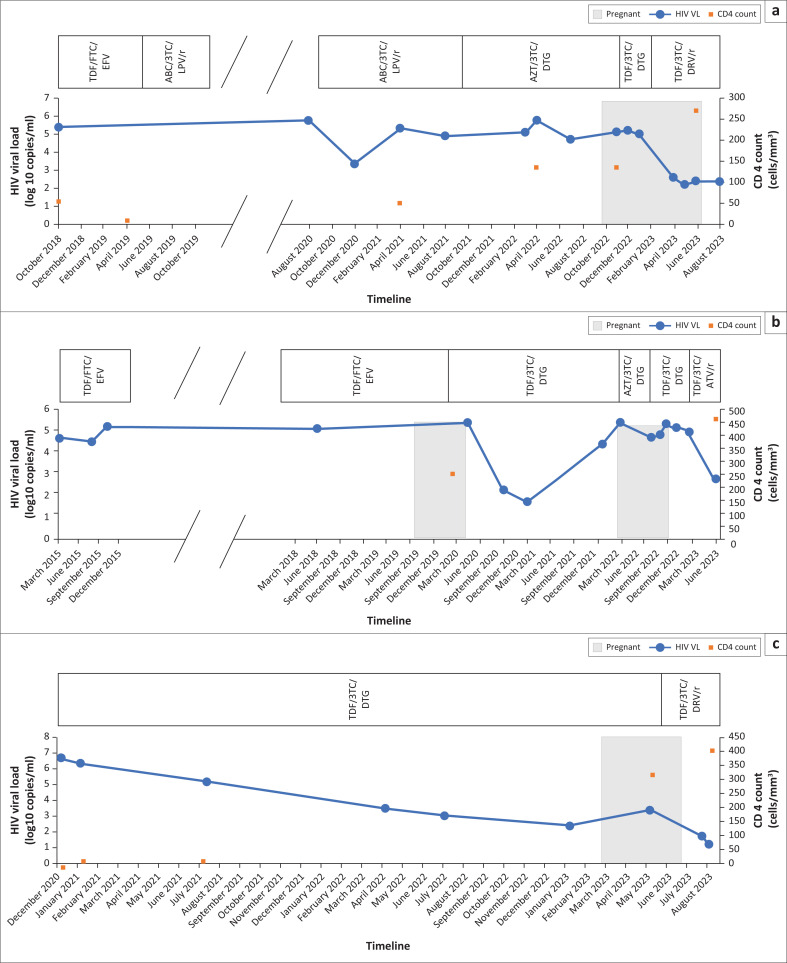
Summary of the HIV viral load, CD4 count and antiretroviral therapy timeline for each case. (a) Case 1. (b) Case 2. (c) Case 3.

Case 1, 32-years-old, was diagnosed with pulmonary tuberculosis (TB) and HIV in 2012. In 2018, with a CD4 count of 6 cells/mm^3^, she developed first-line virological failure and was switched to lamivudine/abacavir/lopinavir/ritonavir. She experienced poor adherence and a seven-month treatment interruption between January 2020 and July 2020, following a bereavement and subsequent depression. After re-engaging in August 2020, she did not achieve viral suppression. In September 2021, with a VL of 91 600 copies/mm^3^ (log 4.96; Figure 1), she was switched to lamivudine/zidovudine/DTG. She continued to experience depression, treatment fatigue, and suboptimal adherence.

In November 2022, at nine weeks’ gestation, she attended her first antenatal care (ANC) visit for an unplanned pregnancy and had a VL of 169 000 copies/mm^3^ (log 5.2). Throughout the pregnancy, she reported improved adherence, with no missed doses, vomiting, or drug-drug interactions. Despite this, repeat VLs remained > 1000 copies/mm^3^. At 22 weeks’ gestation, after 17 months on a DTG-based regimen, HIV drug resistance testing confirmed high-level DTG resistance (Table 1). At 27 weeks’ gestation, she was switched to once-daily emtricitabine/tenofovir/darunavir/ritonavir. Delivery VL was 189 copies/mm^3^ (log 2.2). She was advised to exclusively formula feed and lamivudine/zidovudine/nevirapine infant prophylaxis was provided for six weeks. Both birth and 10-week infant HIV polymerase chain reaction (PCR) tests were HIV–negative.

**TABLE 1:** Summary of confirmed HIV drug resistance mutations and eligibility for HIV resistance testing.

Identified resistance mutations	Case 1	Case 2	Case 3
**Antiretroviral class**			
PI	M46L	None	None
NRTI	M184V	K219R M184V M41L	A62V K65R M184V T215I/T, S68N
NNRTI	K103N	A98G E128A K101H K103N P225H	K103N V106I/M/V
InSTI	E138K G118R T66I	E138K G118R T66A	E138K G118R T66A
**Criteria**			
Classified as TLD2 (second-line TLD)‡	Yes	No *(Only disclosed prior ART exposure after resistance testing)*	No
On TLD for > 24 months	No **(17 months)**	Yes	Yes
2 consecutive VL > 1000 copies	Yes	Yes	Yes
Objective adherence assessed > 80%	Yes (For 3 months before testing)	Yes (For 4 months before testing)	Yes (Throughout ART treatment)
Concurrent TB treatment with unboosted DTG	N/A	N/A	Yes
Met all resistance testing criteria†	No: < 24 months on TLD2	No: classified TLD1	No: classified TLD1

PI, protease inhibitor; NRTI, nucleoside reverse transcriptase inhibitor; NNRTI, non-nucleoside reverse transcriptase inhibitor; InSTI, integrase inhibitor; N/A, not applicable; TLD, tenofovir/lamivudine/dolutegravir; VL, viral load; TB, tuberculosis; DTG, dolutegravir.

† , Based on 2023 South African ART guidelines eligibility criteria.

‡ , TLD2: a regimen is classified as TLD2 if there is a confirmed history of treatment failure on a previous antiretroviral therapy (ART) regimen. A regimen is classified as TLD1 if there is no confirmed history of treatment failure on a previous ART regimen.

### Case 2

In February 2020, Case 2, a 34-year-old woman, presented with an unplanned pregnancy at 22 weeks’ gestation and was newly diagnosed, by self-report, with HIV. She delivered before ART initiation in May 2020, with a VL of 223 000 copies/mm^3^ (log 5.4; Figure 1). The infant was exclusively formula fed and given zidovudine/nevirapine infant prophylaxis cover for six weeks. Both birth and 10-week infant HIV PCR tests were negative. She only initiated TLD in August 2020, with the delay attributed to a combination of psychosocial and systems-related issues. By February 2021, she was virally suppressed, at 50 copies/mm^3^ (log 1.7).

The next VL in February 2022, was 225 000 copies/mm^3^ (log 5.4). In September 2022, a second unplanned pregnancy was confirmed at 23 weeks’ gestation. Responding to the unsuppressed VL of 43 500 copies/mm^3^ (log 4.7), she was switched to zidovudine/lamivudine/DTG. She was then referred to an advanced clinical care clinic where multiple adherence issues were identified and addressed, and she was switched to TLD.

Throughout this pregnancy, and beyond delivery, despite improved adherence of > 80% based on regular clinic attendance, pharmacy refills, and an adequate DTG blood level of 0.979 µg/mL, VLs remained unsuppressed.

The infant was exclusively breastfed and was provided six weeks of zidovudine and 12 weeks of nevirapine prophylaxis. At 12 weeks post-partum, with a VL of 120 000 copies/mm^3^ (log 5.1), she was advised to wean her infant; zidovudine/nevirapine infant prophylaxis was restarted, and resistance testing confirmed high-level DTG resistance (Table 1). The infant’s birth, 10-week, and 6-week post-breastfeeding HIV PCRs were negative.

Only after receiving resistance results did Case 2 disclose additional ART experience prior to 2020, with low adherence and multiple treatment interruptions. A retrospective search of the NHLS database at this point confirmed the prior history. Case 2 reported being initiated on a single daily pill which, based on guidelines from that time, would have most likely been tenofovir/emtricitabine/efavirenz (TEE). Between 2015 and 2020 she reported multiple interruptions but no change in regimen, and VLs from that period were never suppressed. She was switched to tenofovir/emtricitabine/darunavir/ritonavir in June 2023. A repeat VL in September 2023 was < 20 copies/mm^3^ (log 1.2).

### Case 3

In December 2020, Case 3, a 35-year-old woman, was admitted with newly diagnosed TB and HIV. Her new HIV diagnosis was confirmed through a thorough search of the NHLS database, as well as a blood donor card indicating her last recorded donation in late 2018. TLD was initiated two weeks after TB treatment. She was critically ill, with a CD4 count of 2 cells/mm^3^. As well as TB, she had active cytomegalovirus (CMV) infection and herpes simplex virus (HSV)-1 and -2 serology was positive. The authors did not have access to the admission record to confirm whether she received treatment for these two conditions. During TB treatment, DTG was prescribed once-daily, in contradiction to guidelines requiring boosted DTG through 12-hourly dosing to compensate for rifampicin-DTG drug-drug interaction.

Despite being ART naïve, with > 80% adherence (confirmed through regular clinic attendance and pharmacy refills) her VLs remained between 1300 copies/mm^3^ (log 3.1) and 190 335 copies/mm^3^ (log 5.3) (Figure 1), from July 2021 to June 2022. She reported taking over-the-counter ‘immune boosters’ from a pharmacy, the name of which she could not recall. She took these every morning during the period of TB treatment, at which point she was taking TLD at night. She reported no other medication use, including traditional remedies. In January 2023, after stopping the ‘immune boosters’ her VL was 224 copies/mm^3^ (log 2.4).

In May 2023, she attended ANC with an unplanned pregnancy at 26 weeks’ gestation. Her VL was 2 790 copies/mm^3^ (log 3.4), and CD4 count 318 cells/mm^3^. During her pregnancy she vomited most mornings but continued taking TLD at night. Resistance testing was ordered at 27 weeks’ gestation and DTG resistance was confirmed at 29 weeks (Table 1), prompting a switch to once-daily emtricitabine/tenofovir/darunavir/ritonavir. After 1 month, her VL was 59 copies/mm^3^ (log 1.7) and at delivery was 25 copies/mm^3^ (log 1.4). The infant had a negative HIV PCR at birth, was exclusively breastfed, and was provided with zidovudine/nevirapine infant prophylaxis cover.

## Discussion

We have described the first reported cases of DTG resistance in PBFW in the South African ART programme. In all cases, there were intensified clinical support during antenatal and postnatal care, and vertical transmission of HIV was prevented.

In Cases 1 and 2, suboptimal adherence, failure to screen, diagnose and refer for mental health support, poor clinical management of high VLs over prolonged periods, as well as previous ART exposure were likely driving factors for resistance. In Case 3, the lack of DTG boosting during TB treatment is the only identified cause. This is an important observation considering the RADIANT-TB (standard versus double dose dolutegravir in patients with HIV-associated tuberculosis [https://clinicaltrials.gov/study/NCT03851588]) trial reported no significant difference in viral suppression or resistance when DTG was ‘unboosted’ during TB treatment.^14^ One possible reason for Case 3 developing DTG resistance may have been her very high rate of viral replication at ART initiation, with a baseline VL of 5 040 000 copies/mm^3^ (log 6.7), in combination with a very low CD4 count.^15^ Furthermore it is possible that, although she had good support following discharge from hospital, she had suboptimal adherence due to being very unwell and having a significant pill burden.

One of the cornerstones of comprehensive VTP programmes is avoiding unintended pregnancies in women living with HIV.^16^ One survey among pregnant women in South Africa in 2019 showed a 51.5% prevalence of unintended pregnancies in women living with HIV and engaged in care.^17^ It is important to note that all three cases presented with unplanned pregnancies, with Case 2 having two unplanned pregnancies within two years, highlighting an ongoing failure of the South African ART programme to effectively integrate contraceptive services into HIV services for women of reproductive age. Effective integration of reproductive health services with HIV services is particularly important for women experiencing advanced HIV disease and/or unsuppressed VLs because vertical transmission risks are elevated in the context of suboptimal maternal health.

Several key factors influencing ART adherence and VL suppression are well documented in the literature, including medication side effects and pill burden, substance use, presence or lack of social support, comorbid mental health conditions, and duration on treatment.^18,19^ Two of the three cases reported longstanding mental health and psychosocial challenges that persistently undermined their ability to adhere well to their ART regimens and remained unaddressed during years of routine care. This emphasises the importance of integrating psychosocial and mental health support into HIV services. In particular, better integration into antenatal and postnatal care remains a key component of supporting PBFW to achieve and maintain viral suppression during their VTP journey.

These cases demonstrate the difficulties encountered in high-HIV-burden settings, even with supportive clinical and counselling relationships, in achieving full, open discussions about prior ART history, adherence patterns, and other medication use. In the South African public healthcare setting, some information required to satisfy resistance testing criteria can also be complicated to ascertain. Reasons include gaps in the health information systems, including the lack of a universal unique patient identifier. Persons living with HIV also fear stigmatisation upon disclosing treatment interruption. Consequently, patients, like Case 2, often preferentially re-engage as new patients via repeat HIV testing.^20^ It is noteworthy that Case 2 re-engaged with each pregnancy, a common pattern of re-engagement among female patients.^21^ Unfortunately, with increasing reports of DTG resistance,^9,10^ clinicians in South Africa need to be aware that, although DTG is robust, resistance remains a possibility in a small proportion of individuals. This case series adds to the evidence raising concerns that in programmatic settings, DTG resistance may be more frequent than predicted, particularly in contexts where inadequate adherence support and VL monitoring result in prolonged periods of viral non-suppression.

Because DTG has a high resistance barrier, the current South African ART guidelines focus on addressing adherence issues in unsuppressed individuals and provide strict resistance testing criteria. The managing clinicians in these cases, although fully aware of the current guidelines, had specific concerns related to the risk of vertical transmission and used clinical judgement to proceed with drug resistance testing, despite the cases not meeting all the criteria. Strict adherence to the time on ART and previous ART failure (classified as TLD2) criteria for resistance testing, would have delayed identification of resistance, potentially leading to vertical transmission.

### Recommendations

Acknowledging the limitations of a case series, including anecdotal fallacy and lack of generalisability, the authors recommend the following:

- Resistance testing criteria may need to be more flexible, particularly for pregnant and breastfeeding patients. Poor treatment adherence, low CD4 count and high VL at DTG initiation or switch, and drug-drug interactions, are all risk factors for integrase resistance.^15^ All our cases had at least one VL above 100 000 copies/mm^3^ and CD4 < 200 cells/mm^3^ at some point during treatment. Criteria may need to be reviewed for individuals with CD4 < 200 cells/mm^3^ or VLs above 100 000 copies/mm^3^.
- Enhanced programmatic DTG resistance surveillance, considering the high number of individuals in the South African ART programme with suboptimal adherence, low CD4 counts and/or high VL at DTG initiation or switch, or with drug-drug interactions.^22^
- A thorough review of evidence including programmatic data regarding the use of boosted DTG during TB treatment is needed before any future updates are made to clinical guidelines, including antiretroviral and TB guidelines, based on the findings of the RADIANT-TB trial. We agree with Catteano & Gervasoni that the dose of DTG should be doubled when used with rifampicin, particularly in patients with high VLs.^23^
- Improved integration of contraceptive services within the ART programme, with a particular focus on women with unsuppressed VLs and/or advanced HIV disease.
- Inclusion of mental health screening and support for all patients with adherence challenges or treatment interruptions.

While InSTI resistance occurred rarely in clinical trials, it may become more prevalent than anticipated in programmatic settings, especially where adherence and retention support is insufficient. These cases highlight that despite DTG’s high resistance barrier, individuals will still experience resistance. Clinicians should remain alert to the possibility of DTG resistance and programmatic surveillance should be strengthened.
